# Mechanisms of Cancer Inhibition by Local Anesthetics

**DOI:** 10.3389/fphar.2021.770694

**Published:** 2021-12-07

**Authors:** Yiguo Zhang, Yixin Jing, Rui Pan, Ke Ding, Rong Chen, Qingtao Meng

**Affiliations:** ^1^ Department of Anesthesiology, Renmin Hospital of Wuhan University, Wuhan, China; ^2^ Department of Anesthesiology, East Hospital, Renmin Hospital of Wuhan University, Wuhan, China

**Keywords:** local anesthetics, cancer cells, cellular mechanisms, lidocaine, bupivacaine, ropivacaine

## Abstract

The use of local anesthetics during surgical treatment of cancer patients is an important part of perioperative analgesia. In recent years, it has been showed that local anesthetics can directly or indirectly affect the progression of tumors. *In vitro* and *in vivo* studies have demonstrated that local anesthetics reduced cancer recurrence. The etiology of this effect is likely multifactorial. Numerous mechanisms were proposed based on the local anesthetic used and the type of cancer. Mechanisms center on NaV1.5 channels, Ras homolog gene family member A, cell cycle, endothelial growth factor receptor, calcium Influx, microRNA and mitochondrial, in combination with hyperthermia and transient receptor potential melastatin 7 channels. Local anesthetics significantly decrease the proliferation of cancers, including ovarian, breast, prostate, thyroid, colon, glioma, and histiocytic lymphoma cell cancers, by activating cell death signaling and decreasing survival pathways. We also summarized clinical evidence and randomized trial data to confirm that local anesthetics inhibited tumor progression.

## Introduction

The high morbidity and mortality of malignant tumors is a difficult problem for human life and health. Although radical surgery, radiotherapy, chemotherapy, immunotherapy, and hormone therapy are used, the recurrence and metastasis of cancer remain key problems. Surgery is the primary method to treat malignant tumors. However, there is growing evidence that surgical treatment may actually promote cancer recurrence and metastasis. Whether these events occur largely depends on the ability of the tumor to spread and the host’s immune and inflammatory responses.

**FIGURE 1:**
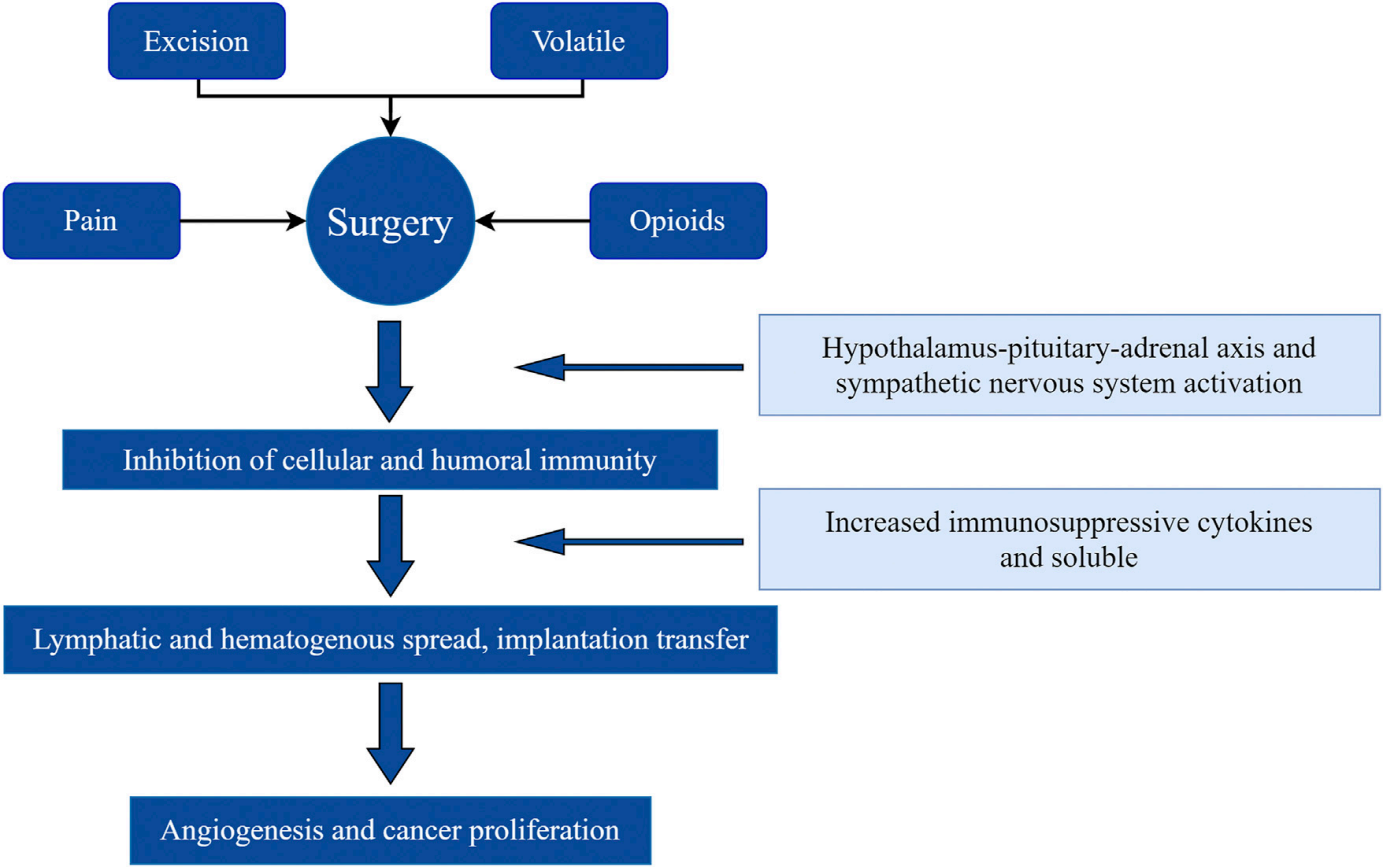
Operative critical factors leading to angiogenesis and cancer cell proliferation.

Surgery provides an opportunity to eradicate tumors, but it also allows residual cancer cells to proliferate and invade. Surgery increases the shedding of malignant cells into the blood and lymph circulation, inhibits their apoptosis, and enhances their invasive ability (Neeman and Ben-Eliyahu, 2013). Surgery also increases the lever of tumor vascular-related and growth factors, and supports local and distant metastasis and tumor recurrence. Significant changes in the immune, endocrine, and inflammatory systems in response to surgery promote cancer progression (Gottschalk et al., 2010). Psychological distress (anxiety, stress, and depression) associated with surgery releases stress hormones, lowers cellular immunity, reduces the host’s immune response, and increases the risk of metastasis (Thornton et al., 2010) (Figure 1).

There is increasing evidence that anesthesia techniques and other perioperative factors potentially influence long-term outcomes after malignant tumor surgery. Local anesthetics (LAs) can inhibit the development of tumors and limit tumor metastasis *via* a variety of mechanisms. Local anesthetics block voltage-gated sodium channels (VGSCs) on nerve cell membranes, and these channels are present on tumor cell membranes and are associated with the invasion and metastasis of tumor cells (Lirk et al., 2012). Local anesthetics have indirect effects on cancer biology. The following anti-tumor mechanisms were proposed(Neeman and Ben-Eliyahu, 2013): anti-tumor cell proliferation and metastasis(Gottschalk et al., 2010), induction of cell apoptosis(Thornton et al., 2010), improvement of chemotherapeutic efficacy(Lirk et al., 2012), reduction of the demand for opioids (Cata et al., 2020). Opioids are immunosuppressive, and their use may reduce a patient’s resistance to tumor metastasis (Dan et al., 2018).

## Overview of Local Anesthetics and Clinical Implications in Cancer Treatment

Since the appearance of cocaine in 1884, LAs have been widely used in all types of surgeries to relieve pain. LAs primarily block voltage-dependent Na^+^ and K^+^ channels, which blocks nerve transmission and produces local anesthesia. The chemical formula of LAs consists of aromatic rings, amino groups and intermediate chains. According to different intermediate chains, LAs divided into esters such as procaine, tetracaine, etc., and amides such as lidocaine, ropacaine, and bupivacaine. LAs may be used alone or in combination with general anesthetics. Combination therapy reduces the dose of general anesthetics, improves the anesthetic effect and reduces the neuroendocrine stress response and perioperative immunosuppression, and may directly inhibit the proliferation and metastasis of tumor cells (Dubowitz et al., 2018).

Thirty female cervical cancer patients who received radical hysterectomy were treated with lidocaine (1.5 mg/kg iv followed by 1.5 mg/kg ivpump and discharge). The interferon-gamma (IFN-*γ*)/Interleukin-4 (IL-4) ratio of the lidocaine group was better than the control group, and the apoptosis of lymphocytes was weaker than the control group. These results suggest that lidocaine has a protective effect on anti-cell-mediated immunity (CMI) in patients with radical hysterectomy of cervical cancer. This treatment may help reduce the incidence of postoperative septic complications and the formation of tumor metastasis (Wang et al., 2015). Lidocaine also decreased the viability of all breast cancer cell lines, inhibited the migration of tumor breast epithelium, and inhibited the immobile growth of triple-negative cells. Intraperitoneal injection of lidocaine improved the survival rate of MDA-MB-231 mice with peritoneal carcinomatosis. The dose of lidocaine is consistent with the current clinical analgesia setting (10 mg/ml) (Chamaraux-Tran et al., 2018).

## Effects of Local Anesthetics on Cancer Cells

### Inhibition of NaV1.5 Channels

Cancer cells and tissues express VGSCs, and VGSCs activity increases the lateral motility and invasion of tumor cells *in vitro* (Fraser et al., 2005). VGSCs play an important role in the occurrence and development of tumors (Pedersen and Stock, 2013; Hofschröer et al., 2020), and functionally expressed in many types of tumor cells (epithelial carcinoma), including breast, cervical, ovarian, prostate, colon, skin, and lung cancers (Fraser et al., 2014). The overexpression of these channels enhances the metastasis cascade and tumor cell metastasis. LAs inhibit VGSCs function, and prevent VGSCs activity during and after surgery, which reduces the ability of cancer cells to escape and metastasize from the perioperative range of surgery. These effects reduce cell proliferation and indirectly increase patient survival.

Ropivacaine inhibited the invasion of SW620 colon cancer cells in a concentration range of 10–100 uM, which was similar to the current effect on the Nav1.5 mutant channel of the neonatal isoform, and this range is related to LAs blockade of sodium channels (Baptista-Hon et al., 2014). Shilpa Dutta et al. found that Nav1.5 was overexpressed in the highly invasive human breast cancer cell line MDA-MB-231. 1uM Nav1.5 blocker inhibited the invasion of MDA-MB-231 cells, and the rate of invasion inhibition was 30.3 ± 4.5%, and fortunately, cell viability was not affected (Dutta et al., 2018). LAs can block the VGSCs which blocks channels in resting, open, and inactivated states (inactivated states have the highest binding affinity) (Grandhi and Perona, 2020). Amide LAS, especially lidocaine, have more systemic anti-inflammatory benefits and effects on immune cells than other LAS agents (Van Der Wal et al., 2015).

The expression of the Nav1.5 can be blocked by Lidocaine in highly metastatic human breast cancer MDA-MB-231 cells (Fraser et al., 2014). Tetrodotoxin (TTX), which is a blocker of VGSCs, was used as a local anesthetic for the treatment of pain in cancer patients in clinical trials, and it showed significant anticancer effects *in vivo* and *in vitro* (Makarova et al., 2019).

### Inhibition of Ras Homolog Gene Family Member A Migration

Rho and RAC GTP enzymes regulate all types of cell migration (Zheng et al., 2020), and one of the activators of the Ras homolog gene family member A (RhoA) pathway is neuroepithelial cell gene 1 (NET1) (Pang et al., 2021). Rho-associated protein kinase (Rock) is a downstream effector of the RhoA pathway. Rock activates myosin phosphatase targeting subunit 1 (MYPT1), and it phosphorylates myosin on myosin light chain 1 (MLC1) (Dan et al., 2018). Low concentrations of bupivacaine (10–50 mM) reduced the migration of gastric cancer cells *via* the RhoA and MLC1 pathways but had no significant effect on tumor growth or survival (Dan et al., 2018). Bupivacaine decreased the phosphorylation of MYPT1 and MLC1, which reduced the migration of gastric cancer cells. Research also suggests that bupivacaine inhibits the migration of cancer cells by stimulating NET1 (Pang et al., 2021). NET1 is significantly up-regulated in gastric and breast cancers, which suggests that bupivacaine is involved in tumor migration *via* this pathway to inhibit tumor migration. Levobupivacaine and ropivacaine reduced tumor cell invasion and migration by reducing RhoA protein levels (Castelli et al., 2020).

Some studies showed that sodium channel blockade contributed to the anticancer activity of LAs (Fraser et al., 2014), but these studies also demonstrated that bupivacaine inhibited the migration of gastric cancer cells *via* the sodium-independent channel blocker RhoA and NET1 inhibition (Dan et al., 2018; Zheng et al., 2020; Pang et al., 2021). These results suggest that LAs are associated with the RhoA and NET1 pathways in addition to sodium channel blockade.

### Cell Cycle

Cell cycle progression is a hallmark of cancer, primarily because Cyclins D1, E, and B2 are key regulators of the cell cycle, and are dysregulated in different cancers, including breast, esophageal, bladder, skin, and lung cancers (Icard et al., 2019). Treatment of breast cancer and melanoma cells with bupivacaine and lidocaine significantly decreased cell cycle proteins, which promoted cell cycle arrest (Castelli et al., 2020). Ropivacaine arrested liver cancer cells in the G2 phase (Le Gac et al., 2017). P53 is a cancer marker that induces cell cycle arrest at G2/M or G0/G1 and activates the expression of the cyclin-dependent kinase inhibitor p21. P27 is a tumor suppressor that regulates the G0 to S phase, and increased P53 phosphorylation (active form) and P27 were observed after treatment with LAs, which indicated impaired cell proliferation.

Golgi apparatus transporter 1A (GOLT1A) is significantly elevated in patients with lung adenocarcinoma, and it was associated with prognosis and pathological staging (Yang et al., 2018a). The down-regulation of GOLT1A inhibits cell proliferation and induces cell cycle arrest. After 2 mmol/L lidocaine for 24 h, the expression of GOLT1A, Cyclin D1, and Cyclin E1 was decreased, which prevented the cells from transitioning from the G1 phase to the S phase (Zhang et al., 2017). Notably, GOLT1A also modulates the sensitivity of breast cancer cells to tamoxifen and improved prognosis (Zhang et al., 2017).

### Epidermal Growth Factor-Associated Effects

Epidermal growth factor receptor (EGFR) is a tyrosine kinase receptor that regulates cell proliferation and differentiation of epithelial cells and tumors, including head and neck, breast, colorectal, lung, and pancreatic cancers (Mitchell et al., 2018). EGFR may be incorrectly activated by a variety of mechanisms: Ligand-dependent dimerization, point mutation, partial deletion, or overexpression. EGFR is expressed in tumor and non-tumor cells in the tumor microenvironment (TME). EGFR plays a role in the stimulation of vascular endothelial growth factor (VEGF), fibroblast growth factor and interleukin-8 (IL-8), which suggests that it supports tumor cell proliferation, angiogenesis, and metastasis (Grapa et al., 2019).

EGFR is activated by specific ligands, such as pro-epidermal growth factor and transforming growth factor-beta (TGF-*β*). Once activated, EGFR dimers stimulate intrinsic protein tyrosine kinase activity in cells, which results in the automatic phosphorylation of several tyrosine residues within EGFR-expressing cells. Therefore, several signal transduction pathways, including mitogen-activated protein kinase (MAPK), protein kinase B (Akt) and c-jun N-terminal kinase (JNK), are initiated, which leads to DNA synthesis and cell proliferation (Sakaguchi et al., 2006). Lidocaine inhibited the proliferation of human tongue cancer cells (CAL27 strain). 400 microM Lidocaine inhibited serum- and EGF-induced CAL27 proliferation *via* inhibition of the auto-phosphorylation of EGFR tyrosine residues without cytotoxicity. With the increase of clinical concentration, 4,000 microM lidocaine inhibited the proliferation of CAL27 cells by inhibiting the activity of EGFR (Sakaguchi et al., 2006).

LAS preferentially induced the EGFR pathway in breast cancer cells (MCF-7) *via* exogenous and endogenous caspase-dependent apoptosis. The activation of EGFR leads to an increase in the downstream activity of caspase 8 and 9, which leads to the apoptosis of breast cancer cells (Li et al., 2018). Treatment of human hepatoma cells (HEP G2 cells) with 1 mM or 5 mM lidocaine showed a continuous increase in caspase 3 concentrations, which reached a maximum level after 24 h (Xing et al., 2017).

### Reduction of Calcium Influx

The increased activity of TME ion channels leads to an increase in intracellular calcium concentration. An increased level of Ca^2+^ in the cytosol promoted the formation of podosomes/invadopodia, which facilitated the invasion of cancer cells (Hantute-Ghesquier et al., 2018). Calcium is also a key regulator of cell invasion. Lidocaine inhibits chemokine-induced tumor cell migration *via* the direct inhibition of CXCR4 activity. Calcium signaling controls the progression and apoptosis of cancer cells *via* the transient receptor potential subfamily member 6 (TRVP6) channel of the transient receptor potential channel V subfamily (Xuan et al., 2016; Liu et al., 2020a). TRVP6 mRNA and protein were detected in ovarian, breast, prostate, thyroid cancer and colon cancers. These results suggest that TRVP6 plays an important role in tumorigenesis, progression and prognosis. After treatment with 100 μm lidocaine for 12 h, the expression of TRPV6 was reduced 50–80%, and the survival rate, migration and cell division of MDA-MB-231 cells were decreased (Xuan et al., 2016). TRPM7 also affects the activity of cancer cells, and it is involved in Ca^2+^ and Mg^2+^ steady-state ion channels (Liu et al., 2020a). Lidocaine inhibited glioma cell proliferation and metastasis *via* the blockade of TRPM7 channels, which prevented the cell cycle and induced protective autophagy (Leng et al., 2017).

### Regulation of microRNA and Mitochondrial Inhibition

MicroRNAs (miRNAs) play important roles in gene silencing and post-transcriptional regulation (Bartel, 2004). Several cancer-related miRNAs were sensitive to LAs *in vitro*, including miR-21, miR-145, miR-520a-3p, and miR-539 (Xuan et al., 2016; Yang et al., 2018b; Yang et al., 2018c; Yang et al., 2019). Lidocaine enhanced the toxicity of cisplatin in lung cancer *via* miR-21 regulation (Xuan et al., 2016), and inhibited the growth, migration and invasion of gastric cancer cells *via* the up-regulation of miR-145 (Sui et al., 2019). Adenosine triphosphate (ATP) levels are strongly associated with tumor cell growth and survival. Mitochondrial metabolism limits tumor proliferation and metastasis by altering ATP levels. Ropivacaine inhibited breast tumor proliferation and metastasis by destroying mitochondrial complexes I and II but not III or IV (Gong et al., 2018). Bupivacaine inhibited mitochondrial complexes I and III to inhibit thyroid tumor proliferation (Chang et al., 2014). Bupivacaine (1 mm and 5 mm) inhibited mitochondrial complexes I and II to induce a decrease in ATP. There was no similar decline in ATP levels or activity after bupivacaine administration in mutant gastric cancer cells without mitochondria (Gong et al., 2018). Bupivacaine inhibits the growth of tumor cells by reducing the level of ATP in mitochondria.

### In Combination With Hyperthermia

Hyperthermia is a non-invasive, localized cancer treatment option that induces targeted cancer cell death. Local hyperthermia induces cell damage in the tumor area with minimal damage to the surrounding tissue (usually 40–44°C) (Markowitz and Bressler, 2021). Under high temperature alone, human histiocytic lymphoma (U937) cells showed a certain degree of DNA fragmentation and nuclear fragmentation, but the degree of nuclear fragmentation was enhanced in a dose-dependent manner when amides were used in combination. LAS with higher liposolubility had a greater promotion effect of heat-induced apoptosis. Intracellular Ca^2+^ concentrations are elevated during high-temperature-induced apoptosis, and the Ca^2+^-chelating agents (BAPTAAM) inhibit DNA fragmentation, which suggests that calcium-dependent pathways are involved in hyperthermia-induced apoptosis (Arai et al., 2002). Moderate temperature (42°C) combined with a low concentration of lidocaine (0.2%) significantly increased skin cancer cell death (Raff et al., 2019). Previous studies also showed that cells in the intermediate and advanced phase of S are more sensitive to high temperatures, with an increased proportion of cells in S phase compared to five types of cancer cells (fibroblasts, keratocytes, melanoma, cervical cancer, basal cell carcinoma) were more sensitive to combination therapy (Raff et al., 2019). The causes of LAS combined with high temperature-induced cancer cell death include the formation of superoxides, a decrease in mitochondrial membrane potential, the activation of caspase 3 and an increase in intracellular Ca^2+^ (Raff et al., 2019).

### Inhibition of Transient Receptor Potential Melastatin 7 Channels

Transient receptor potential melastatin 7 channels (TRPM7) participate in Ca^2+^ and Mg^2+^ steady-state ion balance, which affects cell viability. TRPM 7 is not regulated in many cancers, including head and neck, breast, thyroid, lung, and pancreatic cancers (Grandhi and Perona, 2020). TRPM7 overexpression in bladder cancer cells promoted the proliferation of cancer cells. Regulation of Ca^2+^ homeostasis is associated with cancer development (Gao et al., 2017). Some studies showed the migration of pancreatic cancer cells by TRPM7 *via* the regulation of Mg^2+^-dependent mechanisms (Rybarczyk et al., 2012). Another *in vitro* study showed that TRPM7 was associated with the growth of the human breast cancer cell line MCF-7 (Guilbert et al., 2009). Lidocaine inhibited the proliferation and metastasis of breast cancer cells by inhibiting the function of TRPM7 channels in breast cancer cell lines (Liu et al., 2021). Lidocaine inhibited TRPM7 channels in a concentration-dependent manner. The inhibition rate was 20% at 1 mM and 50% at 3 mM (Leng et al., 2017), but the dose of lidocaine in this study was higher than the clinical dose. Lidocaine prevented the cell cycle and induced protective autophagy in glioma cells by blocking TRPM7 channels (Leng et al., 2017). Another calcium channel, TRPV6, is also expressed in MDA-MB-231 human breast cancer cells, PC-3 prostate cancer cells and ES-2 ovarian cancer cells (Jiang et al., 2016). Lidocaine inhibited the migration and invasion of MDA-MB-231 cells by inhibiting this channel (Jiang et al., 2016).

## Clinical Trials of Local Anesthetics in Tumor Suppression

LAs inhibit the occurrence and development of tumors. Some reliable clinical evidence and randomized trial data also support relevant conclusions. The effect of intravenous lidocaine on tumor-related outcomes after cancer resection was studied using *in vivo* tumor models. Levels of MMP-2, which is a key protein in the metastatic potential of breast cancer cells (Wall et al., 2019) and lung metastasis colonies (Freeman et al., 2019) were reduced after intravenous administration of lidocaine compared to sevoflurane. Lidocaine inhibited tumor growth and increased sensitivity to cisplatin in a xenograft model of hepatocellular carcinoma (Xing et al., 2017). Clinical studies have not investigated the effect of perioperative intravenous lidocaine on the long-term prognosis of cancer. However, Toner et al. showed that intravenous lidocaine was safe, effective and feasible in patients undergoing breast cancer surgery (Toner et al., 2021), and the VAME-C trial (NCT04316013, ∼5,376 patients) is an international randomized controlled trial to compare propofol-TIVA with inhaled sevoflurane and intravenous lidocaine/placebo using a 2 × 2 trial design for colorectal and lung cancer surgery (Dubowitz et al., 2021). These studies should provide high-level evidence for the significant role of lidocaine in tumor anesthesia. A few clinical studies investigated the effects of local anesthesia and intravenous lidocaine on the post-operative inflammatory response. It is suggested that intravenous lidocaine during the procedure until 1 h postoperatively can decrease the inflammatory cytokine (IL-1, IL-6, IL-10, TNF-*α*, and IFN-*γ*) and increase the anti-inflammatory cytokine (IL-10) (Ortiz et al., 2016). The Association for the Promotion of Postoperative Recovery guidelines for perioperative care during elective colorectal surgery strongly recommends lidocaine infusion during colorectal surgery (Gustafsson et al., 2019). However, intravenous lidocaine has a risk of toxicity, and guidelines and rational perioperative administration are essential.

The preliminary evidence is compelling, but there is insufficient high-quality evidence to fully explain the role of LAs in tumor regulation and support changes in current clinical practice. With some large prospective trials underway, our understanding of the impact of anesthesia on cancer-related outcomes should improve rapidly in the future. There will also be stronger evidence that LAs inhibit tumor progression, and relevant clinical guidelines will be developed to provide best practice guidelines for tumor anesthesia and treatment.

## Discussion

**FIGURE 2:**
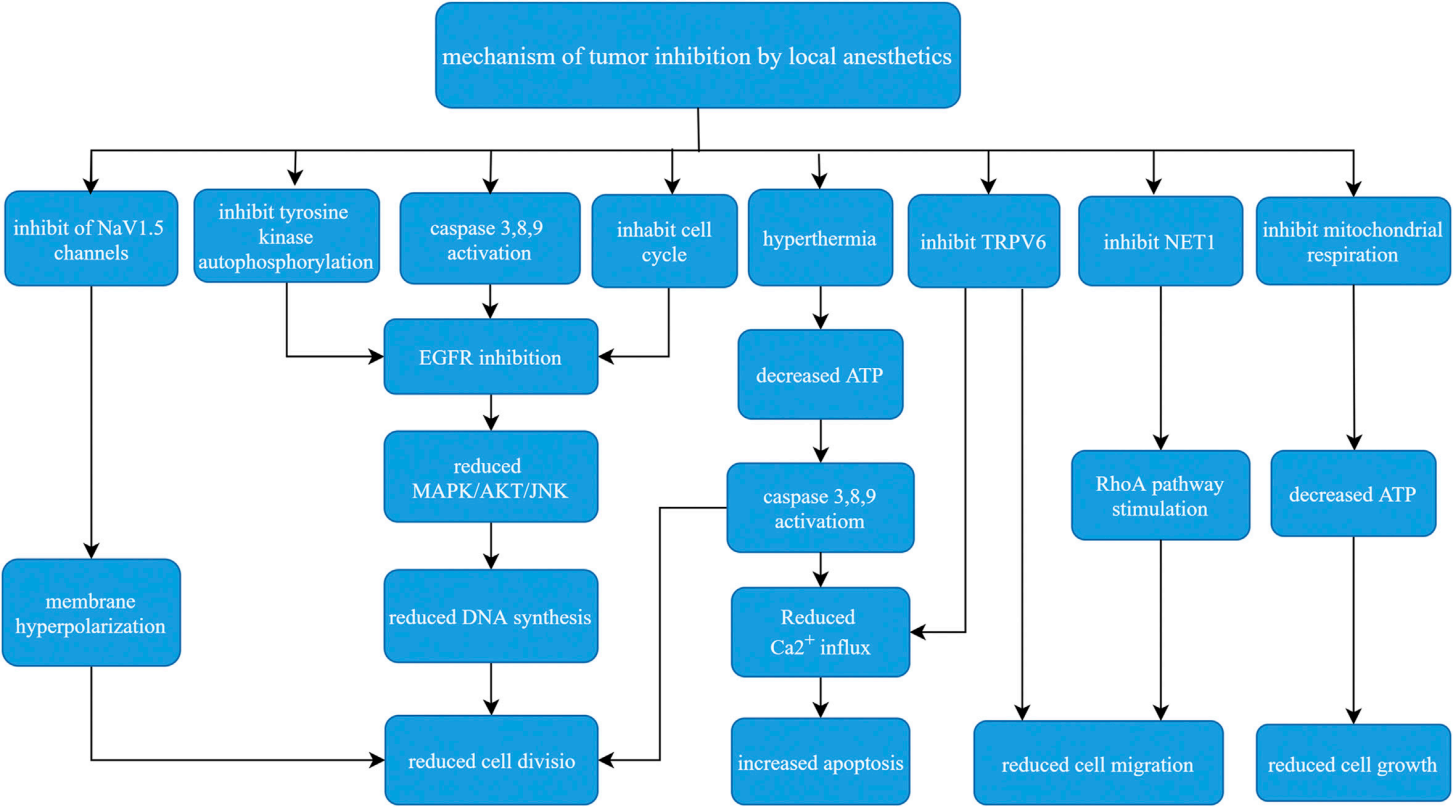
Mechanism of local anesthetics inbiting cancer cells.

Evidence is mounting to address the effects of anesthesia, anesthetics, anesthesia techniques, and surgical stress on long-term cancer outcomes. Surgery remains one of the main treatments of tumors, especially early benign tumors. It is obvious that the choice of anesthesia methods and anesthetics are key to the treatment of cancer. Current research on LAs in oncology suggests that the role of LAs is not independent, and these agents are more likely to be used as chemosensitizers or synergistic therapies. For example, lidocaine increases the sensitivity of breast cancer cells to tamoxifen by down-regulating the expression of GOLT1A, which enhanced prognosis (Zhang et al., 2017), Lidocaine also enhances the toxicity of the cancer drugs mitomycin C, pirarubicin, softening lotion and cisplatin (Sui et al., 2019; Gong et al., 2018). This article reviewed the basic pharmacology of LAs, their mechanisms of action on tumor cells, and their current clinical application. LAs primarily inhibit the proliferation and metastasis of tumor cells, induce apoptosis, improve the efficacy of chemotherapy, and reduce the need for opioids to fight against cancer. These mechanisms interact to form a local anesthetic anti-tumor mechanism network (Figure 2). Liu et al. showed that all LAs were toxic to cancer cells at high concentrations, but different anesthetics have different effects, and the same tumor cell line had a different local anesthetic, such as bupivacaine > lidocaine > ropivacaine (Liu et al., 2020b). Laboratory and human studies showed that lidocaine reduced the levels of the tumor markers IL-1, TNF-*α* and IL-8 and had a direct effect on cancer cells *via* blockade of voltage-gated sodium channels or other mechanisms. Lidocaine reduced the viability and migration of cancer cells in laboratory studies and increased the survival rate of breast cancer mice (Li et al., 2018).

Despite extensive experimental evidence of the potentially beneficial effects of the perioperative use of regional and LAs, the exact role and impact of the use of these substances in cancer surgery are not clear. The drug concentrations in many animal and cell experiments were significantly higher than clinical use (Leng et al., 2017), but there were also consistent results with clinical concentrations (Chamaraux-Tran et al., 2018). There is still a lack of support from clinical data from randomized controlled trials. Studies *in vitro* and animal models do not always fit human clinical conditions perfectly. There is growing evidence that different types of anesthetics promote or inhibit metastasis, depending on the type of tumor cell and the type, dose and regimen of anesthetics used. Continuous intravenous infusion of lidocaine during the perioperative period has been safely used to reduce systemic inflammation and intestinal dysfunction (Gustafsson et al., 2019), after abdominal surgery, and the results of this study also provide a basis and guidance for the role of LAs in tumor therapy. Although lidocaine is currently a local anesthetic that may be administered intravenously and is most widely used in anesthesiology clinics, it has not always been the most effective anticancer agent *in vitro* studies. Intravenous lidocaine is also not without risk. Perhaps future studies focusing on the *in vivo* and *in vitro* effects and mechanisms of certain types of tumors may yield better results in the pharmaceutical field for the development of new intravenous LAs with high anti-tumor effects and low toxicity as a more attractive solution.

Future research into the effects and mechanisms of LAs on cancer cells is promising and necessary. This review boldly conceived and summarized some of the solutions and research priorities:

1) More vivo studies. We encourage the addition of more animal experiments in this area. Animal experiments are closer to the reality of the human body than cell experiments. These studies simulate human cancer surgery conditions in animal models and evaluate tumor suppression, metastasis, and related endpoints for animal survival, which will help elucidate the systemic effects of LAs.
2) Standardization of experimental methods. There is no consistency between research groups on the effects of LAs on cancer cells. The reason for the inconsistent results may be that the experimental methods have not been well standardized or due to some experimental difficulties caused by the drugs themselves, such as the low solubility of some LAs and local anesthetic toxicity during intravenous administration. Appropriate controls and standardization are required to eliminate the potential effects of different experimental protocols to produce consistent and repeatable results.
3) Investigations of the effects of LAs on tumor stem cells. The presence of tumor stem cells raises the question of whether these cells or differentiated cancer cells drive tumorigenesis (Dawood et al., 2014). Previous studies showed that lidocaine, ropivacaine and bupivacaine were effective inhibitors of leukemic stem cell colony formation, and non-cancer stem cells are unaffected by these LAs (Ni et al., 2018). Although these studies are sporadic, they provide a reference for the treatment of tumors and a feasible idea for the mechanism of LAs in inhibiting the development of tumors. If LAs are found to have a more consistent ability to interfere with cancer stem cells, the widely studied differences between differentiated cancer cell types may be reconciled.
4) Investigation of the specificity of tumors. It is generally difficult to kill most tumors *via* the intravenous administration of drugs at the site of surgery or at the time of surgery. Specific tumors or types of cancer cells that may be particularly sensitive to certain LAs should be identified. It is even possible to compare the effects of LAs on cancer and non-cancer cells to help determine the specificity of LAs.
5) Use bioinformatics. Advanced gene sequencing techniques and bioinformatics tools may be applied to problems related to LAs and tumors. This use may help identify multiple mechanisms of tumorigenesis and reveal new targets of LAs. Identification of the correlation between the two may lead to a better solution for the treatment of tumors.
